# Geographical Variation in the Associations Between School Characteristics and Homophobic Bullying: a Contextual Analysis

**DOI:** 10.1007/s11121-024-01732-4

**Published:** 2024-09-23

**Authors:** Salvatore Ioverno, Amy McCurdy, Stephen T. Russell

**Affiliations:** 1 Department of Education, Roma Tre University, Via del Castro Pretorio 20, 00185 Rome, Italy; 2 Texas Higher Education Coordinating Board, 1801 Congress Ave. Suite 12.200, Austin, TX 78701 USA; 3 Department of Human Development and Family Sciences, University of Texas at Austin, 108 E Dean Keeton St., Stop A2702, Austin, TX 78712 USA

**Keywords:** Victimization, Homophobic bullying, School characteristics, Geographic variation, Contextual influences

## Abstract

Homophobic bullying constitutes a serious threat to adolescent well-being and could be understood as an ecological phenomenon, influenced by diverse school, regional, and community contexts. This study examined geographic variations in the relationship between school characteristics and homophobic bullying. Data from 2244 California schools, including student surveys and administrative records, were combined and analyzed using geographically weighted regression (GWR). Results showed that the associations between school characteristics and general victimization were consistent across geographic areas. However, when it came to homophobic bullying, the relationships with school characteristics varied significantly based on location. Notably, regions with high intolerance, urbanity, large school size, and small student–teacher ratios appeared to offer protection against bullying. Additionally, student socioeconomic status influenced bullying in disadvantaged rural schools with limited Gay-Straight Alliance (GSA) presence. Ethnic diversity also played a role, with low diversity or dominance of two ethnic groups linked to higher bullying rates.

**Supplementary Information:**

The online version contains supplementary material available at 10.1007/s11121-024-01732-4.

## Introduction

Sexual and gender minority students face an elevated risk of being targets of homophobic bullying, a form of victimization based on actual or perceived sexual orientation or gender identity (Moyano & del Mar Sánchez-Fuente, 2020; Myers et al., 2020; Turanovic et al., 2019). The prevalence of this form of bullying varies significantly, ranging from 4 to 50%, as indicated by a recent systematic review (Moyano & del Mar Sánchez-Fuente, 2020). Similar to general bullying, homophobic bullying is linked to adverse mental health and academic consequences (Baiocco et al., 2015; Bishop et al., 2021; Pistella et al., 2019, 2020). However, evidence suggests that homophobic bullying uniquely contributes to more severe outcomes for students, surpassing the impact of general victimization (Myers et al., 2020).

Previous studies often overlooked the geographic context in examining school characteristics and bullying, despite the inherent ties between schools and their communities (Muijs, 2017; Saarento et al., 2015a, b; Saarento et al., 2015a, b). Identifying local characteristics strongly associated with bullying incidents is essential for effective policy strategies. This study investigates geographic variations in both homophobic bullying and the potential association between geographic patterns of school characteristics and bullying. Additionally, the analysis explores geographic variation in general victimization, recognizing potential differences in correlates compared to homophobic bullying.

### Contextual Correlates of Victimization and Homophobic Bullying

Limited prior research has explicitly explored geographic variability in homophobic bias-based bullying, despite numerous studies indicating context-dependent differences in bullying rates (Muijs, 2017; Saarento et al., 2015a, b; Saarento et al., 2015a, b). Such contexts are more than simply a backdrop against which homophobic bullying occurs; the characteristics of schools and communities may be factors associated with bullying or with education, intervention, or prevention efforts. Several school-level characteristics may be particularly relevant: urbanicity and school size; student–teacher ratio and the presence of experienced teachers; racial/ethnic, socioeconomic, and LGBTQ diversity; and school climate and student organizations like Gender-Sexuality Alliance clubs (GSAs). Importantly, these school characteristics both co-occur and vary geographically. Given a dearth of research on school-level characteristics that are associated with homophobic bullying, we discuss associations with general victimization in the following section—we note links with homophobic bullying when available.

#### Urbanicity and School Size

Urban schools, typically larger than rural schools, are generally expected to have higher bullying rates, but empirical evidence shows mixed results (Saarento et al., 2015a, b; Turanovic et al., 2019). Bradshaw et al. (2009) found that suburban schools had higher bullying rates than rural schools, but no differences were observed between urban and rural schools. Klein et al. (2010) found that rural schools, many of which are small and do not have transitions between grades (which might dislodge existing social hierarchies), actually had higher bullying prevalence relative to urban schools. In one study (Saarento et al., 2015a, 2015b), students in larger schools reported feeling more alienated and disconnected, potentially contributing to higher bullying rates. However, the association between student enrollment numbers and bullying is inconclusive. While some studies found a positive link between school size and bullying (Gregory et al., 2010), others did not (Klein et al., 2010; Saarento et al., 2015a, b; Turanovic et al., 2019), suggesting that other factors, such as school policies (Muijs, 2017), may play a stronger role in predicting bullying. In one study (Klein et al., 2010), the rate of bullying experiences per student was actually higher in smaller schools, challenging the assumption that larger schools always have more bullying cases.

#### *S*tudent–Teacher Ratio and Experienced Teachers

The student–teacher ratio is an important indicator of available time and resources for student supervision and is associated with less bullying. Studies show a positive link between student–teacher ratios and bullying incidents, with schools having higher ratios experiencing more bullying (Bradshaw et al., 2009). Additionally, experienced teachers are more likely to employ effective anti-bullying interventions, working with both perpetrators and victims (Burger et al., 2015).

#### Student Diversity

The diversity of students in schools, including racial/ethnic, socioeconomic, and sexual orientation diversity, is relevant to bullying. Some studies suggest that greater racial/ethnic diversity may lead to conflict and oppression (Jansen et al., 2016), while others argue it reduces prejudice and bullying (Closson et al., 2014). The association between ethnic diversity and bullying varies across studies, with some finding a positive link and others a negative link (see Basilici et al., 2022 for a review). Klein et al. (2010) distinguish between the proportion of students from minoritized ethnic backgrounds and overall ethnic diversity. In schools with a larger proportion of ethnic minority students, teacher-reported bullying is higher, while greater ethnic diversity is linked to less bullying. Basilici et al. (2022) emphasize the contextual aspect, noting that studies in North America, compared to Europe, show a negative association between school ethnic diversity and bullying, especially in high schools.

Schools with more students from low socioeconomic status (SES) backgrounds have been found to have higher rates of bullying (Azeredo et al., 2015; Gregory et al., 2010; Klein et al., 2010). This link is often measured by the percentage of students eligible for free or reduced-price meals. The explanation for this association is partly attributed to students from low SES backgrounds living in high-crime neighborhoods (Dietrich & Zimmermann, 2019). However, one study suggests that the relationship between SES and bullying is fully mediated by the quality of student–teacher and student–student relationships (Dietrich & Cohen, 2021).

Few studies have examined the connection between the proportion of LGBTQ students in schools and school-level homophobic bullying. Given that LGBTQ students are more targeted for such bullying (Bucchianeri et al., 2016), schools with a higher percentage of LGBTQ students may report increased instances. While LGBTQ students are more prone to non-biased-based victimization than cisgender heterosexual students (Myers et al., 2020), the specific links between the proportion of LGBTQ youth in schools and both general victimization and homophobic bullying are still not thoroughly explored.

#### School Climate

School climate is a multidimensional concept encompassing perceptions and feelings of students, teachers, and personnel regarding connections, values, teaching quality, and safety within schools (Ioverno & Russell, 2022). It significantly affects student well-being, making it a crucial predictor and correlate of bullying and victimization within schools (Turanovic et al., 2019). On one hand, bullying strongly impacts student perceptions of safety at school (Ioverno & Russell, 2020). On the other hand, positive school climates, particularly those that promote diversity and respect (Gage et al., 2014), are associated with reduced victimization and declining bullying rates.

#### Gender and Sexuality Alliances (GSAs)

GSAs are student-initiated school clubs that have the primary purpose of promoting a positive school climate and student culture through respect for all students, regardless of their sexual orientation and gender identity or expression (Ioverno & Russell, 2020). At the school level, the presence of GSAs has been linked to positive school climate and other school-related constructs for both LGBTQ and cisgender, heterosexual students. A meta-analysis found that GSA presence in schools was associated with less homophobic victimization and greater perceived safety among high school students (Marx & Kettrey, 2016). Relevant to the current study, the presence of a GSA club has been linked with other school characteristics: schools with GSAs tend to have higher student enrollment, greater ethnic diversity, a lower proportion of low SES students, and a higher proportion of LGBTQ students (Baams et al., 2018). Moreover, GSAs’ effectiveness may depend on the quality of the school climate (Ioverno & Russell, 2020).

### Current Study

Homophobic bullying is an ecological phenomenon influenced by diverse contexts (Basilici et al., 2022; Ioverno, 2023). Research on school characteristics and homophobic bullying, excluding consistent findings for school climate and GSA clubs, has produced mixed results, possibly due to overlooked geographic variation. California, with its diverse student population and urbanicity, is strategically important for this investigation. Prior large-scale studies on homophobic bullying in California schools provide a foundation for understanding the effects of bullying on student well-being and school climate (Ioverno & Russell, 2020, 2022; Ioverno et al., 2022). This study aims to complement existing findings by examining geographic diversity.

The study aims to identify spatial patterns of school characteristics and explore how regional variability in these characteristics relates to rates of homophobic bullying, using general victimization for comparison. Cluster analyses categorize schools based on local regression patterns, and geographically weighted regression (GWR) is employed to examine spatial variations in associations between school characteristics and general victimization/homophobic bullying.

The study aims to identify spatial patterns of school characteristics and explore how regional variability in these characteristics relates to rates of homophobic bullying, using general victimization for comparison. Geographically weighted regressions (GWR) were employed to examine spatial variations in associations between school characteristics and general victimization/homophobic bullying, and cluster analyses categorized schools based on local regression patterns.

## Methods

### Data Sources

Three data sources were merged for this study. Student reports on victimization, bullying, school climate, and sexual/gender identities were from the California Healthy Kids Survey (CHKS). Administered biennially in public middle and high schools by WestEd with support from the California Department of Education (CDE), CHKS surveys covered 7th-, 9th-, and 11th-grade classrooms. The second source comprised publicly available school characteristics from the CDE. Third, the Genders and Sexualities Alliance Network provided GSA presence data. Combining CDE data from 2015, CHKS data from 2014 to 2015, and GSA census data from 2015, the final sample included 2244 schools, about 21% of California schools in 2014–2015.

### Measures

#### General Victimization and Homophobic Bullying

General victimization was assessed using a 9-item scale (Ioverno et al., 2022; *α* = 0.82), assessing physical and verbal assault and harassment on school property within the past 12 months. Responses, ranging from 0 (0 times) to 3 (4 or more times), were dichotomized and summed due to skewness and kurtosis, resulting in scores from 0 to 9. For homophobic bullying, CHKS survey participants reported occurrences in the past 12 months. Response options, from 0 (0 times) to 3 (4 or more times), were dichotomized (0 = no report; 1 = report). Using these two scales, aggregate mean scores were calculated for each school, reflecting both the average number of victimization experiences and the percentage of students reporting incidents of homophobic bullying.

#### School Characteristics

The California Department of Education (CDE) classified schools’ urbanity into urban areas (i.e., areas of 50,000 or more people), urban clusters (i.e., areas of at least 2,500 and less than 50,000 people), and rural areas (i.e., all population, housing, and territory not included within an urban area). CDE also provides public online access to databases on demographic indicators by school: student enrollment, pupil/teacher ratio, average years of teaching by teachers, and percentage of socioeconomically disadvantaged students (receipt of free or reduced-price meal). The CDE calculates ethnic diversity with higher values indicating a more even distribution of students across eight race/ethnicity categories (see http: //www. cde.ca.gov/ds/).

#### School Climate

The CHKS school climate measure comprised fifteen indicators across four sub-constructs: caring relationships with adults, perceived school safety, meaningful participation, and school connectedness. Response options ranged from 0 (strongly disagree) to 4 (strongly agree) for school connectedness and the first school safety item, and from 0 (not at all true) to 3 (very true) for caring relationships with adults and students’ meaningful participation. One safety item had five response options from “Very safe” to “Very unsafe.” In line with Ioverno and Russell (2022), a comprehensive scale integrating the four dimensions was created using the standardized *z*-scores of each item, where higher scores indicate positive climates.

#### Percentage of LGBT Students

In the CHKS survey, students were asked: “Which of the following best describes you? Mark all that apply.” Response options included “Heterosexual;” “lesbian, gay, bisexual;” “transgender;” “unsure;” and “decline to respond.” Two dichotomous variables were created: LGBT and non-LGBT. Based on this item, a percentage of LGBT students was calculated for each school.

#### GSA Presence

Schools were coded for the presence of a GSA based on data from the Genders and Sexualities Alliance Network (GSA Network), which maintains a California statewide registry of GSAs (http://gsanetwork.org/).

#### Geographic Data

The geographic coordinates for each school were provided by the CDE (2015). The 2015 TIGER/Line shapefiles provided geographical boundaries of the regions in the state of California, which we used to create the maps and conduct analyses.

### Statistical Analyses

First, ordinary least squares (OLS) regression assessed associations between school characteristics and rates of general victimization and homophobic bullying. Subsequently, geographically weighted regression (GWR) models were employed to investigate spatial heterogeneity. GWR, generating multiple regressions for each observation, allows exploration of spatial variation in the relationship between the dependent variable and predictors. Coefficient estimation employs weighted least squares, giving greater influence on geographically closer observations. GWR models yield a distribution of local estimated parameters (adjusted *R*^2^, intercepts, slopes, and standard residuals) rather than single parameters for each variable. The weighting scheme depends on bandwidth, the distance from an observation beyond which the weight becomes 0. An adaptive kernel function, sensitive to observation density, was used, enhancing accuracy in predictions for sparsely distributed points over large distances.

The Global Moran’s index identified spatial autocorrelation in the standard residuals of the OLS models, testing the null hypothesis that beta coefficients are randomly distributed geographically. A significant positive index indicated spatial clustering, while a significant negative index suggested spatial dispersion of high or low associations between predictors and dependent variables. Furthermore, comparisons of the Akaike information criterion (AIC) and variance accounted for by each model (*R*^2^) were conducted to assess OLS and GWR model performance, with lower AIC and higher *R*^2^ values indicating better performance.

Finally, a two-step cluster analysis categorized spatial coefficients estimated using GWR, classifying schools into areas where general victimization and homophobic bullying were homogeneously associated with school-level predictors. The best cluster solution was chosen based on an average silhouette measure of cohesion and separation greater than 0.2 and the lowest value of Schwarz’s Bayesian Criterion (BIC) with a ratio of BIC changes between six and ten and the maximum ratio of distance measures across the different cluster classifications (Bacher et al., 2004). For ease of interpretation, we describe the clusters based on the most prominent beta coefficients, i.e., those that were significant in all or the majority of the schools within the clusters and whose effect size was significantly higher than the state-level average. In addition, the interpretation of the cluster based on the distribution of the beta coefficients was coupled with the analysis of the general descriptive statistics (chi-square and ANOVAs) of the key variables for each cluster.

## Results

The table in the supplemental materials shows descriptive statistics for all key variables in this study. OLS regression models are presented in Table 1. Results showed that schools in rural areas with high pupil/teacher ratios tended to have higher rates of general victimization, and schools with higher enrollment tended to have lower rates of homophobic bullying. Ethnic diversity and the percentage of students receiving FRPM were associated with higher victimization and homophobic bullying, whereas the percentage of LGBT students was associated with less general victimization but more homophobic bullying. Positive school climate and the presence of GSAs were associated with less general victimization and homophobic bullying. The models explained 15% of the variance in general victimization and 12% of the variance in homophobic bullying.

**Table 1:** Ordinary least squares regression results for homophobic bullying and general victimization

	General victimization				Homophobic bullying		
	*B*	*SE*	*β*	*B*		*SE*	*β*
Intercept	1.78	0.08		6.43		0.65	
Rural areas	0.33	0.05	0.13***	0.32		0.40	0.02
Urban cluster	0.05	0.03	0.03	0.04		0.26	0.00
Enrollment	0.00	0.00	− 0.03	0.00		0.00	− 0.07*
Pupil/teacher ratio	0.01	0.00	0.09***	0.02		0.02	0.03
*M* years of teaching	0.00	0.00	0.01	0.02		0.03	0.01
Ethnic diversity	0.01	0.00	0.24***	0.05		0.01	0.19***
% FRPM	0.00	0.00	0.14***	0.01		0.00	0.06*
% LGBT students	− 0.02	0.00	− 0.13***	0.17		0.02	0.16***
School climate	− 0.10	0.02	− 0.15***	− 0.85		0.13	− 0.16***
GSA presence	− 0.31	0.03	− 0.25***	− 1.82		0.25	− 0.19***
*R*^2^	0.16			0.12			
Adjusted *R*^2^	0.15			0.12			
AICc	3267.44			12,610.25			

*p < .05; ***p < .001; *GSA* Gender-Sexuality Alliance, *AIC* Akaike’s information criterion; FRPM indicates the percent of students eligible for free or reduced-price meals. For the measure of ethnic diversity, higher values indicate more evenly distributed students among eight race/ethnicity categories

For general victimization, Moran’s I was not significant, *I* = 0.21; *z* = 1.72; *p* = 0.085, indicating no spatial clustering of the standard residuals. In contrast, a positive and significant Moran’s I was found for homophobic bullying, *I* = 0.40; *z* = 3.26; *p* = 0.001, indicating geographically clustered patterns of association and providing evidence for preferring a GWR model over an OLS model. Lower AIC and higher *R*^2^ in the GWR model compared with the OLS model confirmed the improved model performance.

Table 2 shows the estimated coefficients of the GWR model for homophobic bullying. Higher enrollment, positive school climate, and presence of a GSA were consistently and negatively associated with homophobic bullying across regions of California, whereas ethnic diversity, the percentage of students with FRPM, and the percentage of LGBT students were positively associated. Other school characteristics varied in the direction and strength of their link with homophobic bullying in different regions.

**Table 2:** Geographically weighted regression results for homophobic bullying

	Coefficient range					
	Min	1st quartile	Median	3rd quartile	Max	% significant coefficients
Rural areas	− 0.18	− 0.04	0.03	0.08	0.38	9.63%
Urban cluster	− 0.29	− 0.06	0.00	0.06	0.33	9.31%
Enrollment	− 0.26	− 0.12	− 0.10	− 0.08	− 0.02	22.59%
Pupil/teacher ratio	− 0.09	0.00	0.08	0.13	0.28	26.38%
*M* years of teaching	− 0.18	− 0.06	− 0.03	0.03	0.22	13.46%
Ethnic diversity	− 0.15	0.13	0.16	0.24	0.41	92.38%
% FRPM	− 0.06	0.02	0.04	0.07	0.35	12.92%
% LGBT students	0.02	0.13	0.17	0.21	0.38	83.82%
School climate	− 0.31	− 0.25	− 0.22	− 0.19	0.00	86.14%
GSA presence	− 0.27	− 0.22	− 0.14	− 0.11	0.02	71.17%
Local *R*^2^	0.07	0.13	0.15	0.20	0.33	
*R*^2^	0.21					
Adjusted *R*^2^	0.17					
AICc	6031.07					

*GSA* Gender-Sexuality Alliance, *AIC* Akaike’s information criterion; FRPM indicates the percent of students eligible for free or reduced-price meals. For the measure of ethnic diversity, higher values indicate more evenly distributed students among eight race/ethnicity categories. % significant coefficients indicates the percentage of beta coefficients significant at *p* < 0.05

### Cluster Analysis on Geographically Weighted Regression Coefficients

The nine-cluster solution showed a better fit than solutions with more or fewer clusters (the highest mean distance ratio value compared to the other solutions and an average total silhouette dissimilarity value of 0.6, indicating a good quality classification). Descriptive characteristics of the geographic clusters are presented first, followed by the presentation of the results of the GWR.

#### Descriptive Statistics of Geographic Clusters

A series of chi-squares and ANOVAs were conducted to test whether the means and frequencies of the key variables in each cluster were significantly different from the state average (see Table 3). Cluster 1 had significantly higher rates of homophobic bullying, higher enrollment, and percentage of students receiving FRPM. Cluster 2 included predominantly large urban schools and teachers with high average years of experience. Cluster 3 had fewer schools with GSA and higher ethnic diversity. Cluster 4 included mostly small rural schools without GSAs, with teachers with low average years of experience, low ethnic diversity, and high percentages of students receiving FRPM. Cluster 5 included mostly urban schools with GSAs, low levels of homophobic bullying, high ethnic diversity, a low student–teacher ratio, low average years of teaching experience, and low percentages of students receiving FRPM. Cluster 6 included mainly large schools with teachers with high average years of experience and a high student–teacher ratio. Cluster 7 included mostly small rural schools with low percentages of LGBT students and low student–teacher ratios. Cluster 8 included mostly urban schools, low ethnic diversity, and high teaching experience averages. Finally, cluster 9 included mainly small, non-urban schools with high levels of homophobic bullying, high ethnic diversity, and low percentages of students receiving FRPM.

**Table 3:** Descriptive statistics for each cluster

Clusters	Homoph. bullying	Rural areas	Urban cluster	Urban areas	Enroll	Pupil/teacher ratio	*M* years teaching	Ethnic diversity	% FRPM	% LGBT students	School climate	GSA presence
	*M* (SD)	*N* (%)	*N* (%)	*N* (%)	*M* (SD)	*M* (SD)	*M* (SD)	*M* (SD)	*M* (SD)	*M* (SD)	*M* (SD)	*N* (%)
Cluster 1	**10.73 **^**↑**^	6	18	112	**1256.93 **^**↑**^	23.16	11.26	35.43	**68.42 **^**↑**^	5.58	− 0.18	37
	**(4.67)**	(4.41)	(13.24)	(82.35)	(816.90)	(3.76)	(2.89)	(14.02)	**(22.69)**	(3.80)	(0.60)	(27.21)
Cluster 2	9.20	12	**13 **^**↓**^	**195 **^**↑**^	1069.28	21.49	**12.46 **^**↑**^	36.63	58.41	5.24	0.12	50
	(4.18)	(5.45)	**(5.91)**	**(88.64)**	(751.97)	(4.89)	**(3.49)**	(16.67)	(26.07)	(4.31)	(0.72)	(22.73)
Cluster 3	9.99	**3 **^**↓**^	30	154	849.91	22.81	11.01	**41.58 **^**↑**^	64.40	5.76	− 0.10	**31 **^**↓**^
	(4.52)	**(1.60)**	(16.04)	(82.35)	(637.34)	(3.80)	(2.83)	**(14.04)**	(20.57)	(4.53)	(0.78)	**(16.58)**
Cluster 4	9.74	**20 **^**↑**^	**61 **^**↑**^	**104 **^**↓**^	**730.54 **^**↓**^	22.35	**10.58 **^**↓**^	**23.94 **^**↓**^	**73.99 **^**↑**^	4.66	− 0.06	**25 **^**↓**^
	(5.34)	**(10.81)**	**(32.97)**	**(56.22)**	**(546.51)**	(11.33)	**(3.25)**	**(14.19)**	**(21.13)**	(4.37)	(0.91)	**(13.51)**
Cluster 5	**8.62 **^**↓**^	**3 **^**↓**^	**11 **^**↓**^	**379 **^**↑**^	903.81	**20.14 **^**↓**^	**10.10 **^**↓**^	**42.43 **^**↑**^	**47.32 **^**↓**^	5.48	0.05	**147 **^**↑**^
	**(4.08)**	**(0.76)**	**(2.80)**	**(96.44)**	(667.80)	**(4.36)**	**(3.07)**	**(16.24)**	**(28.64)**	(3.66)	(0.85)	**(37.4)**
Cluster 6	9.38	**2 **^**↓**^	**0 **^**↓**^	265	**1314.65 **^**↑**^	**23.95 **^**↑**^	**12.5 **^**↑**^	32.58	54.70	4.69	0.00	73
	(3.08)	**(0.75)**	**(0.00)**	(99.25)	**(825.87)**	**(3.86)**	**(2.37)**	(16.75)	(29.03)	(3.43)	(0.71)	(27.34)
Cluster 7	9.76	**42 **^**↑**^	**69 **^**↑**^	**90 **^**↓**^	**531.71 **^**↓**^	**19.22 **^**↓**^	10.79	38.11	56.89	**3.51 **^**↓**^	0.18	45
	(4.60)	**(20.90)**	**(34.33)**	**(44.78)**	**(473.12)**	**(4.27)**	(3.28)	(11.16)	(19.95)	**(4.42)**	(0.91)	(22.39)
Cluster 8	8.95	**5 **^**↓**^	**39 **^**↓**^	**387 **^**↑**^	1074.60	21.87	**12.42 **^**↑**^	**27.09 **^**↓**^	62.58	5.36	− 0.07	116
	(4.07)	**(1.16)**	**(9.05)**	**(89.79)**	(775.09)	(4.27)	**(3.41)**	**(15.96)**	(27.07)	(3.55)	(0.79)	(26.91)
Cluster 9	**10.24 **^**↑**^	**19**^**↑**^	**65**^**↑**^	**140 **^**↓**^	**751.76 **^**↓**^	21.10	11.43	**42.41 **^**↑**^	**51.06 **^**↓**^	5.93	− 0.08	49
	**(4.11)**	**(8.48)**	**(29.02)**	**(62.5)**	**(573.32)**	(3.70)	(3.24)	**(15.46)**	**(22.80)**	(4.89)	(0.80)	(21.88)
State-level	9.43	112	306	1,826	955.84	21.66	11.45	35.33	58.35	5.17	0.01	573
	(4.26)	(4.99)	(13.64)	(81.37)	(728.60)	(5.33)	(3.26)	(16.66)	(26.48)	(4.08)	(0.80)	(25.53)

*GSA* Gender-Sexuality Alliance; FRPM = the percent of students eligible for free or reduced-price meals. For the measure of ethnic diversity, higher values indicate more evenly distributed students among eight race/ethnicity categories. Means or percentage in bold are significantly different from the state-level at *p* < .05 (with Bonferroni correction)

^**↑**^Mean or percentage significantly higher than the state-level

^**↓**^ Mean or percentage significantly lower than the state-level

BIC =  − 11,0454.952; a ratio of BIC changes =  − 413.39; maximum ratio of distance measures = 1.33; total silhouette dissimilarity = 0.6

#### Cluster Analysis on Geographically Weighted Regression Coefficients

For enhancing data visualization clarity, the figure in the supplemental materials shows the maps of the nine clusters categorized into three groups based on descending values of the local *R*^2^s (i.e., high, medium, and low). Table 4 reports the mean of all beta coefficients and the percentage of significant beta coefficients. Results showed that the regressions within clusters 1, 2, 3, and 5, on average, explained a larger portion of the variance compared to others. Specifically, the variables included in the regressions within these clusters, on average, accounted for between 20 and 24% of the variance. For ease of interpretation, we describe the clusters by the most prominent beta coefficients, that is, those that were significant in all or most of the schools within the clusters and whose effect size was statistically higher than the state average.

**Table 4:** Mean and percentage of significant beta coefficients for each cluster associated with homophobic bullying rates

Clusters	Statistics	Rural areas	Urban cluster	Enroll	Pupil/teacher ratio	*M* years teaching	Ethnic diversity	% FRPM	% LGBT students	School climate	GSA presence	Local *R*^2^
Cluster 1	Mean	**0.28**	0.09	** − 0.15**	0.11	0.01	**0.28**	0.08	**0.25**	− 0.17	− 0.14	0.24
	% Signif. Coeff	**88.97%**	0.74%	**94.85%**	25.74%	0.00%	**99.26%**	0.00%	**100.00%**	52.21%	58.09%	
Cluster 2	Mean	0.07	0.16	− 0.07	0.10	0.03	**0.33**	− 0.01	**0.19**	** − 0.21**	** − 0.26**	0.21
	% Signif. Coeff	19.55%	0.00%	0.00%	1.82%	0.00%	**100.00%**	0.00%	**100.00%**	**89.09%**	**100.00%**	
Cluster 3	Mean	− 0.06	0.00	− 0.12	**0.16**	0.08	0.19	0.09	**0.26**	** − 0.22**	− 0.15	0.21
	% Signif. Coeff	1.60%	0.53%	0.00%	**93.05%**	28.34%	98.93%	12.83%	**100.00%**	**99.47%**	59.36%	
Cluster 4	Mean	− 0.06	− 0.14	− 0.18	− 0.04	0.06	**0.29**	**0.29**	0.10	** − 0.24**	− 0.04	0.14
	% Signif. Coeff	0.00%	89.19%	66.49%	5.41%	31.35%	**94.05%**	**98.38%**	30.81%	**100.00%**	3.78%	
Cluster 5	Mean	0.00	− 0.01	− 0.09	0.10	− 0.05	0.16	0.06	**0.20**	** − 0.27**	** − 0.22**	0.20
	% Signif. Coeff	0.00%	0.00%	0.00%	27.23%	0.00%	100.00%	11.70%	**100.00%**	**100.00%**	**100.00%**	
Cluster 6	Mean	0.11	0.18	** − 0.12**	0.02	− 0.03	0.16	0.03	0.12	** − 0.23**	− 0.11	0.15
	% Signif. Coeff	4.49%	10.86%	**86.52%**	0.00%	0.00%	100.00%	0.00%	56.55%	**100.00%**	90.26%	
Cluster 7	Mean	− 0.07	− 0.04	− 0.09	0.12	− 0.05	0.05	0.01	0.09	** − 0.21**	** − 0.18**	0.11
	% Signif. Coeff	17.91%	0.00%	0.00%	32.34%	0.00%	44.78%	0.00%	40.80%	**95.52%**	**92.54%**	
Cluster 8	Mean	0.05	− 0.08	− 0.07	− 0.02	− 0.08	0.16	0.05	0.16	− 0.20	− 0.13	0.13
	% Signif. Coeff	0.00%	3.02%	5.57%	0.00%	30.16%	99.77%	8.82%	100.00%	99.30%	82.37%	
Cluster 9	Mean	0.01	0.00	− 0.12	**0.18**	0.08	0.14	0.05	**0.19**	− 0.06	− 0.07	0.11
	% Signif. Coeff	0.45%	0.00%	0.00%	**87.95%**	27.23%	79.91%	0.00%	**100.00%**	6.70%	2.23%	
State-level	Mean	0.03	0.01	− 0.10	0.07	− 0.01	0.19	0.06	0.17	− 0.21	− 0.15	0.16
	% Signif. Coeff	9.63%	9.31%	83.82%	92.38%	26.38%	12.92%	13.46%	22.59%	86.14%	71.17%	

*GSA* Gender-Sexuality Alliance; FRPM = the percent of students eligible for free or reduced-price meals. For the measure of ethnic diversity, higher values indicate more evenly distributed students among eight race/ethnicity categories. Coefficients in bold indicate beta coefficients significant in all or most of the schools within the clusters and whose effect size was significantly higher than the state average

In cluster 1 (which had the highest levels of homophobic bullying), homophobic bullying was higher in schools that were rural and that had low student enrollment, high ethnic diversity, and a high percentage of LGBT students. In cluster 2, homophobic bullying was higher in schools with high ethnic diversity, low school climate, few GSAs, and a high percentage of LGBT students. Schools in cluster 3 with high rates of homophobic bullying had lower school climate scores, a higher percentage of LGBT students, and a higher student–teacher ratio. In cluster 4, homophobic bullying was higher in schools with low school climate scores, high ethnic diversity, and a high percentage of students receiving free meals. In cluster 5, differences between schools were largely due to school climate, few GSAs, and a higher percentage of LGBT students. In cluster 6, the highest levels of homophobic bullying were found in schools with lower enrollment and low school climate. Cluster 7 was similar to cluster 5 (negative school climate and few GSAs), except that the differences were not associated with the percentage of LGBT students. In cluster 8, several factors were associated with homophobic bullying in most schools (lack of GSAs, poor school climate, a higher percentage of LGBT students, and greater ethnic diversity), although effect sizes were not statistically different from the state average. Finally, in cluster 9, schools with higher homophobic bullying were higher in schools with a higher student–teacher ratio and percentage of LGBT students.

## Discussion

This study explored geographic variations in the links between school characteristics and rates of homophobic bullying in California schools, using general victimization as a comparison. Notably, associations of school characteristics with general victimization were consistent across the state, but associations with homophobic bullying varied geographically (GWR model outperforming OLS). In some clusters, school characteristics explained over 20% of the variance in homophobic bullying. Findings underscore the significance of contextual influences contributing to homophobic bullying in schools. Indeed, certain school characteristics carry greater weight than others in elucidating variations in rates of homophobic bullying among schools across different geographic areas.

### General Victimization

Findings regarding general victimization align consistently with prior research (Klein et al., 2010; Saarento et al., 2015a, b; Yoon, 2004): enrollment and teachers’ years of experience showed no significant associations with general victimization. General victimization rates were higher in rural schools compared to urban schools, a pattern noted in prior research (see Saarento et al., 2015a, b for a review) potentially linked to community norms, barriers to bullying prevention, and limited extracurricular activities (Leadbeater et al., 2013). The positive association between student–teacher ratios and general victimization aligns with studies highlighting challenges in addressing bullying within larger classroom settings (Bradshaw et al., 2009).

Ethnically diverse schools and those with higher proportions of students receiving free or reduced meals experienced more general victimization. This contrasts with some studies in California (Bellmore et al., 2012), possibly influenced by measurement differences. Like prior research (Azeredo et al., 2015; Gregory et al., 2010), this study found a link between a high proportion of socioeconomically disadvantaged students and school victimization rates. This correlation may be attributed to the heightened likelihood that socioeconomically disadvantaged students face instances of hate crimes (Dietrich & Zimmermann, 2019) or employ interpersonal aggression for status gain (McAra & McVie, 2016).

Interestingly, a higher percentage of LGBT students correlated with lower general victimization rates, potentially indicating aggressors may selectively target LGBT minority students (Ioverno et al., 2021), leading to lower overall victimization rates due to concentrated aggression. In other words, LGBT minority students may stand out among their peers, becoming targets for aggressors aiming to assert social dominance. The lower rates of victimization observed might stem from the concentration of aggressive behaviors directed at a minority subset of students, possibly experiencing victimization from multiple perpetrators (DeKeseredy et al., 2021).

Finally, a positive school climate and the presence of Gender and Sexuality Alliances (GSAs) were associated with lower rates of general victimization, aligning with multiple previous studies (Turanovic et al., 2019).

### Homophobic Bullying

The study identified statewide school characteristics linked to homophobic bullying. Geographically weighted findings reveal that the overall associations of school characteristics with homophobic bullying differ across regions. Specifically, certain characteristics, such as urbanicity, school size, student–teacher ratio, and student-economic disadvantage, had significant associations in a minority of schools. On the other hand, ethnic diversity, percentage of LGBT students, school climate, and GSA presence exhibited significant associations across the majority of schools, albeit with varied statistical patterns within geographic clusters.

#### Geographic Variability of Correlates of Homophobic Bullying

In contrast to prior studies (Goodenow et al., 2006; Kazyak, 2011), OLS models found no significant rural–urban differences in homophobic bullying. GWR analyses showed significant urbanicity associations in less than 10% of schools, suggesting nuanced impacts. Notably, differences in homophobic bullying between urban and nonurban schools were prominent in regions with higher homophobic bullying rates, economically disadvantaged students, and high enrollment (e.g., cluster 1 around Los Angeles). This finding underscores the importance of prioritizing prevention efforts in areas with structural challenges for addressing homophobic bullying in schools; for example, LGBT youth in rural areas may face a higher risk of school victimization due to conservative values (Wike et al., 2021). Consequently, it is imperative that prevention initiatives extend to the community level, focusing on raising awareness about the issues surrounding homophobic bullying.

According to the GWR model, negative associations between enrollment and homophobic bullying rates were especially notable in regions with higher concentrations of large schools compared to the state average (i.e., in and around Los Angeles County). These findings further support previous research (Goodenow et al., 2006) showing that students are at greater risk for homophobic bullying in smaller schools compared to larger ones.

Although OLS regression did not show associations between student–teacher ratio and homophobic bullying, the GWR model showed significant associations in 26.38% of schools. A higher number of students per class emerges as a risk factor for homophobic bullying in two distinct areas: one marked by schools with limited GSAs (cluster 3) and another characterized by small rural schools with high rates of homophobic bullying (cluster 9). Notably, both areas exhibit high levels of ethnic diversity. In such contexts, teachers with larger student loads may encounter multiple demands, potentially limiting their ability to effectively manage classrooms and, consequently, address homophobic bullying. Prevention efforts could focus on training and support for teachers to build classroom management skills, while school leadership could focus on strategies to reduce class sizes. Additionally, promoting resources such as GSAs can help mitigate the risks associated with high student loads for teachers (Marx & Kettrey, 2016).

The OLS results confirmed a significant negative association between the percentage of students receiving free or reduced-price meals and homophobic bullying rates, aligning with the idea that high-poverty schools may lack resources to combat discrimination against LGBT students (Kosciw et al., 2009). Yet, the GWR model identified this association in only 12.92% of schools. Notably, disparities were concentrated in a specific geographic area characterized by the highest average percentage of disadvantaged students (73.99%) attending predominantly small rural schools with limited presence of GSAs (cluster 4, situated in the Central San Joaquin Valley). These data suggest that schools with high poverty rates might have few resources to implement programs aimed at reducing discrimination against LGBT students (Kosciw et al., 2009). Prevention strategies could include community support and resources to fund GSAs and other strategies to improve school climate, such as training staff on LGBT-inclusive policies.

#### Consistent Correlates of Homophobic Bullying Across Different Geographic Areas

The GWR model identified a significant association between ethnic diversity and homophobic bullying in 92.38% of schools, with notable variation across clusters. Clusters 1, 2, and 4 displayed stronger-than-average associations, even though cluster 4, with the highest association, had the lowest mean level of ethnic diversity in the state. Clusters 1 and 2 had mean levels of ethnic diversity at the state midpoint, indicating a prevalence of two primary ethnic groups (35.43% and 36.63%). Conversely, clusters with the highest mean levels of ethnic diversity exhibited relatively smaller associations. These findings suggest that homophobic bullying tends to be more prevalent in schools with either low ethnic diversity or a balanced representation of two ethnic groups, fostering a potential “us versus them” mentality that may foster misunderstanding, eventually resulting in biased behavior. In contrast, exposure to diverse cultures may mitigate prejudiced attitudes, including those related to sexual orientation (Bellmore et al., 2012). In such settings, bullying prevention efforts could take an intersectional approach, focusing on recognizing and challenging bias based not only on homophobia but also on racism.

OLS findings align with prior studies, indicating that a positive school climate correlates with reduced homophobic bullying (Ioverno & Russell, 2020). However, the GWR model revealed regional variation; regions with high rates of homophobic bullying showed fewer significant differences (e.g., cluster 1 with 52% significant beta coefficients and cluster 9 with 7% significant beta coefficients). On one hand, in environments with high levels of homophobic bullying, the protective impact of a positive school climate might weaken. On the other hand, students may be less inclined to utilize school resources or seek support from peers and teachers when facing homophobic bullying (Ioverno & Russell, 2022). Prevention efforts to reduce homophobic bullying aimed at improving the school climate may be less effective in areas characterized by greater intolerance towards sexual and gender minorities. In such areas, community-level prevention strategies could involve community political and religious leaders sharing messages of tolerance and acceptance in order to improve the broader community climate.

Finally, the OLS model indicated lower rates of homophobic bullying in schools with GSAs, consistent with studies associating GSA presence with reduced bullying risk and enhanced student safety (Marx & Kettrey, 2016). However, the GWR model revealed significant regional disparities in the protective association of GSAs for homophobic bullying. The most pronounced associations were observed in clusters 2, 5, and 7, while a minority of coefficients were significant in clusters 1, 3, 4, and 9. These findings suggest that GSAs may be less effective in combatting homophobic bullying in areas characterized by intolerance towards sexual and gender minorities (i.e., clusters 1 and 9) or with a limited number of GSAs (i.e., clusters 3 and 4). Promoting support for GSAs in regions where their presence is limited could be effective for mitigating rates of homophobic bullying, acknowledging that local attitudes toward LGBT issues can impact the functioning of GSAs (Poteat et al., 2015). Here again, community-level interventions may provide a larger web of support for GSAs.

## Limitations

There are several limitations related to measurement. As is often the case in studies using large data sets (e.g., Seil et al., 2014), measurement limitations include the use of a single-item measure for homophobic bullying in the CHKS, lacking details on its source and severity. Additional items could enhance results for tailored intervention. The omnibus variable for sexual and gender minority identity might obscure within-group variability, particularly regarding transgender or questioning status.

While it is unlikely that bullying shapes most of the school characteristics studied here, the cross-sectional data limits addressing temporal associations. Longitudinal studies are essential for understanding these associations. Also, beyond GSAs, research identifies other strategies (e.g., school policies, teacher development, LGBTQ-inclusive curricula) to improve school climate (Ioverno, 2023). Future studies should explore geographic variability in policies and practices associated with student victimization and bullying. While the geographic focus on California is novel, results may not generalize to other contexts. Future research should investigate geographic variability in the link between school characteristics and student victimization and bullying in different states and countries.

## Conclusions

Factors that predict general victimization among youth are consistent statewide, underscoring the efficacy of universal prevention programs. Yet associations between school characteristics and homophobic bullying vary geographically, indicating the need for contextually sensitive homophobic bullying prevention efforts.

Twenty-five years ago, the state of California passed one of the first LGBTQ-inclusive nondiscrimination laws pertaining to public education in the United States. Our study highlights that even in the context of inclusive education policies statewide, there is contextual variability in factors related to homophobic bullying. Results point to strategies for tailoring school-level prevention, including factors consistent with prior research, such as taking intersectional approaches to promote positive school climates and supporting GSA clubs. At the same time, results also point to the importance of promoting community-level interventions in settings where there may be less tolerance towards sexual and gender minority people. Areas characterized by high enrollment, economic disadvantage, and moderate to low racial and ethnic diversity may be places where effective school-based strategies to reduce homophobia are under-resourced or have weaker effects. In such settings, school-community partnerships to promote school funding and resources, along with messages of inclusion and acceptance in media and from community leaders, could be effective in shifting community norms to reduce intolerance and promote LGBTQ inclusion.

## Supplementary Information

Below is the link to the electronic supplementary material.

Supplementary file1 (DOCX 588 KB)

## Data Availability

"Data will be made available on request."
