# Supplementary material for: Geographical Variation in the Associations Between School Characteristics and Homophobic Bullying: a Contextual Analysis
Source: Prev Sci. 2024 Sep 23;25(7):1164–76. doi: 10.1007/s11121-024-01732-4 (PMC11519247; doi:10.1007/s11121-024-01732-4)
Supplement: Supplementary file 1 — Supplementary file1 (DOCX 588 KB) [file 11121_2024_1732_MOESM1_ESM.docx]

| *Supplemental Table*. Descriptive statistics of the key variables in sample schools (*N* = 2,244) and the remaining California Department of Education (CDE) Schools (*N* = 8,109). | | | | | | | |
| --- | --- | --- | --- | --- | --- | --- | --- |
|  | Sample schools | | | | | | CDE schools |
|  |  | Quartiles | | | | |  |
| Key variables | *M(SD)/ N (%)* | Min | 1^st^ quartile | Median | 3^rd^ quartile | Max | *M(SD)/N(%)* |
| General Victimization | 2.25(0.54) | 0.57 | 1.90 | 2.21 | 2.59 | 5.00 | - |
| *%* homophobic bullying | 9.43(4.26) | 0.00 | 6.85 | 8.88 | 11.29 | 40.00 | - |
| Rural areas | 112(4.99%) | 0.00 | 0.00 | 0.00 | 0.00 | 1.00 | 535(6.62%) |
| Urban clusters | 306(13.64%) | 0.00 | 0.00 | 0.00 | 0.00 | 1.00 | 909(11.24%) |
| Enrollment | 955.84(728.60) | 11.00 | 434.00 | 778.00 | 1255.25 | 4814.00 | 504.40(421.11) |
| Pupil/teacher ratio | 21.66(5.33) | 1.80 | 19.50 | 22.20 | 24.40 | 167.50 | 21.18(8.23) |
| *M* years of teaching | 11.45(3.26) | 1.00 | 9.00 | 12.00 | 14.00 | 27.00 | 11.90(5.13) |
| Ethnic diversity | 35.33(16.66) | 0.52 | 23.03 | 37.67 | 47.01 | 75.26 | 32.77(17.58) |
| % FRPM | 58.35(26.48) | 0.00 | 37.21 | 62.77 | 80.65 | 100.00 | 59.87%(30.16%) |
| % LGBT students | 5.17(4.08) | 0.00 | 2.50 | 4.61 | 7.13 | 36.36 | - |
| School climate | 0.01(0.80) | -2.98 | -0.53 | -0.06 | 0.55 | 3.08 | - |
| GSA presence | 573(25.53%) | 0.00 | 0.00 | 0.00 | 1.00 | 1.00 | 288(3.38%) |
| *Note.* GSA= Gender-Sexuality Alliance; FRPM indicates the percent of students eligible for free or reduced-price meals. For the measure of ethnic diversity, higher values indicate more evenly distributed students among eight race/ethnicity categories. Information regarding general victimization, the prevalence of homophobic bullying, the percentage of LGBT students, and school climate, is sourced from the CHKS surveys, as these data points were not available in the CDE database and GSA census. | | | | | | | |


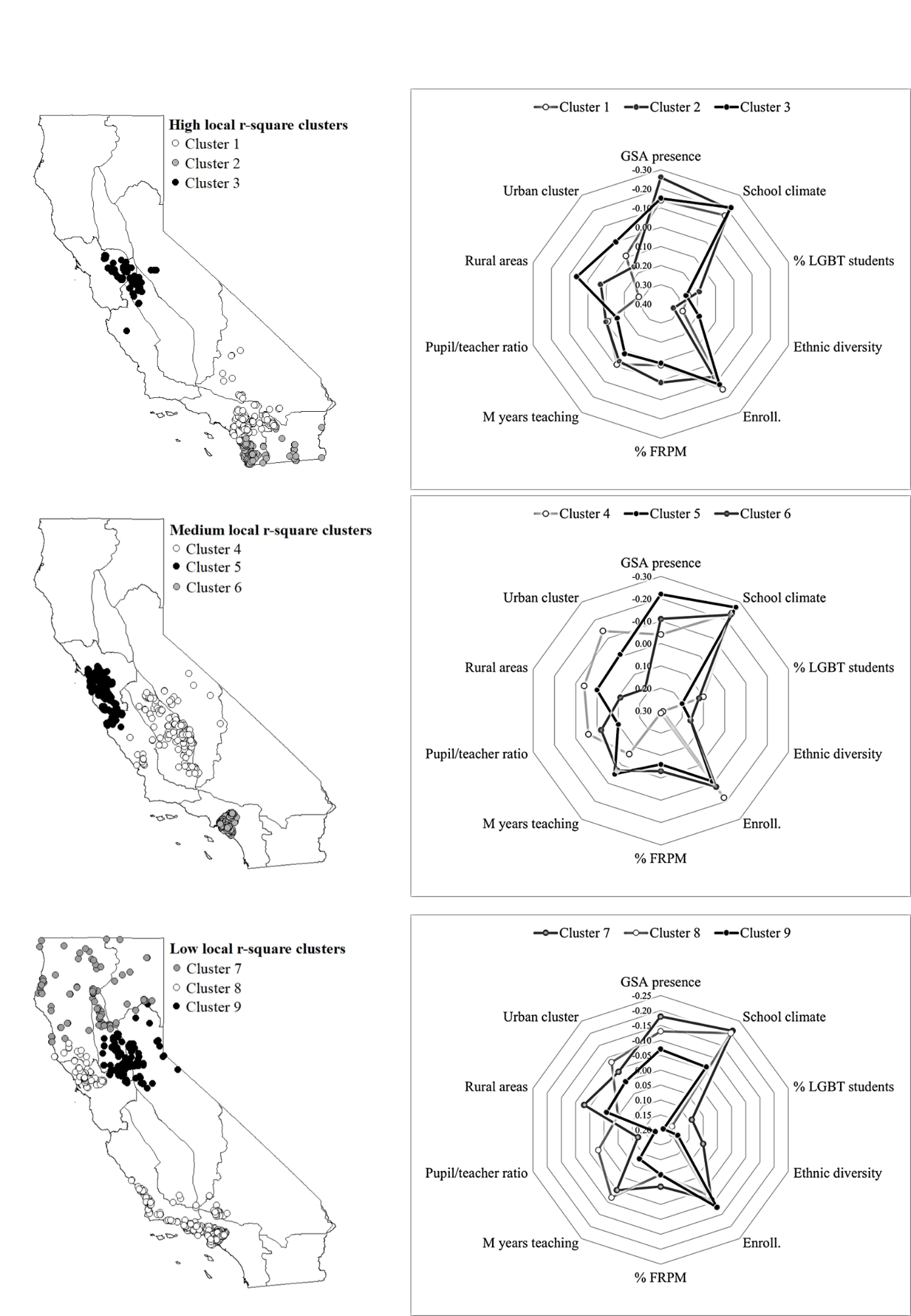


*Supplemental Figure.* Maps and radar plots of clusters based on geographically weighted beta coefficients.
